# West Nile Virus Infection: A Cross-Sectional Study on Italian Medical Professionals during Summer Season 2022

**DOI:** 10.3390/tropicalmed7120404

**Published:** 2022-11-28

**Authors:** Matteo Riccò, Alessandro Zaniboni, Elia Satta, Silvia Ranzieri, Milena Pia Cerviere, Federico Marchesi, Simona Peruzzi

**Affiliations:** 1 Occupational Health and Safety Service on the Workplace/Servizio di Prevenzione e Sicurezza Ambienti di Lavoro (SPSAL), Department of Public Health, AUSL-IRCCS di Reggio Emilia, 42122 Reggio Emilia, Italy; 2 Department of Medicine and Surgery, University of Parma, 43126 Parma, Italy; 3 Università Cattolica del Sacro Cuore, 00168 Rome, Italy; 4 Laboratorio Analisi Chimico Cliniche e Microbiologiche, Ospedale Civile di Guastalla, AUSL-IRCCS di Reggio Emilia, 42016 Guastalla, Italy

**Keywords:** West Nile fever, West Nile virus, knowledge, risk perception

## Abstract

West Nile virus (WNV) has progressively endemized in large areas of continental Europe, and particularly in Northern Italy, in the Po River Valley. During summer season 2022, Italy experienced an unprecedented surge in incidence cases of WNV infections, including its main complications (West Nile fever (WNF) and West Nile neuroinvasive disease (WNND)). As knowledge, attitudes, and practices (KAP) of medical professionals may be instrumental in guaranteeing a prompt diagnosis and an accurate management of incident cases, we performed a cross-sectional study specifically on a sample of Italian medical professionals (1 August 2022–10 September 2022; around 8800 potential recipients). From a total of 332 questionnaires (response rate of 3.8%), 254 participating medical professionals were eventually included in the analyses. Knowledge status of participants was unsatisfying, as most of them exhibited knowledge gaps on the actual epidemiology of WNV, with similar uncertainties on the clinical features of WNF and WNND. Moreover, most of participants substantially overlooked WNV as a human pathogen when compared to SARS-CoV-2, TB, and even HIV. Interestingly, only 65.4% of respondents were either favorable or highly favorable towards a hypothetical WNV vaccine. Overall, acknowledging a higher risk perception on WNV was associated with individual factors such as reporting a seniority ≥ 10 years (adjusted odds ratio [aOR] 2.39, 95% Confidence interval [95%CI] 1.34 to 4.28), reporting a better knowledge score (aOR 2.92, 95%CI 1.60 to 5.30), having previously managed cases of WNV infections (aOR 3.65, 95%CI 1.14 to 14.20), being favorable towards a hypothetic vaccine (aOR 2.16, 95%CI 1.15 to 4.04), and perceiving WNV infections as potentially affecting daily activities (aOR 2.57, 95%CI 1.22 to 5.42). In summary, substantial knowledge gaps and the erratic risk perception collectively enlighten the importance and the urgency for appropriate information campaigns among medical professionals, and particularly among frontline personnel.

## 1. Introduction

West Nile virus (WNV) is a mosquito-borne RNA virus (genus Flavivirus, family *Flaviviridae*) that, since 1962 and similarly to other arboviruses (e.g., tick-borne encephalitis virus or TBEV) [1,2], has progressively become endemic in large areas of continental Europe [3,4,5,6,7,8], where it is mainly carried by endogenous mosquito species of the genus *Culex* (*C. pipiens*, *C. peregrinus*, and *C. modestus*) [7,9,10], with a more limited role for *Aedes* spp. [11,12,13]. Arthropod vectors then sustain the enzootic “amplification” cycle within the main hosts, migratory birds [5,6,7,9,14]. Even though WNV can infect mammalians, including humans, and particularly large mammalians such as horses, the latter only represent incidental and dead-end hosts, as the viremia that WNV infections can reach in mammalians is insufficient to sustain an amplification cycle [9]. Nonetheless, interhuman transmission has been reported, but only through blood transfusion or organ transplants, with significant consequences from a public health point of view, but a limited role on the epidemiology of WNV infections [8,9,15,16,17,18,19].

Human WNV infections usually follow the bite of infected mosquitos, exhibiting a distinctive seasonal trend from April to November, which mirrors the ecology of hosts and vectors [20,21]. Most of cases are usually asymptomatic; however, according to available statistics, up to 20% of them may develop a mild influenza-like syndrome: West Nile fever (WNF) [22,23]. Eventually, an even lesser share of WNV infection cases (around 1% of all cases) may develop a neuroinvasive disorder, i.e., West Nile neuroinvasive disease (WNND), whose main risk factors are represented by belonging to older age groups, being affected by chronic diseases such as solid tumors and chronic kidney disease, and immunodeficiency [22,23,24,25]. 

After the first outbreak in the Tuscany region in 1998, WNV subsequently re-emerged in eight provinces from three regions of Northern Italy (i.e., Lombardy, Emilia-Romagna, and Veneto) during 2008 [19,26,27,28,29]; and since 2010, Northern Italy has become one of the most heavily affected areas in the European Union, with new cases of WNV/WNF and WNND regularly reported every year [8,19,30,31]. Geographic and climatic factors that are particularly favorable to the mosquito vectors of WNV are commonly present in large areas of Northern Italy, and particularly in the Po River Valley, an area including 5 out of 20 Italian regions (Piedmont, Lombardy, Veneto, Emilia-Romagna, and Friuli-Venezia Giulia), and around 41.9% of total Italian population [19,32]. The high occurrence of WNV infections has therefore prompted the implementation of the National WNV Surveillance Plan, which consists of environmental and epidemiological surveillance by integrating veterinary, entomological, and epidemiological data, and includes the routinary screening of WNV in blood samples for transfusion [10,26,27]. The underlying framework of the National Surveillance Plan is guaranteeing the early identification of WNV in the environment [33,34,35], among suitable vectors (i.e., mosquitoes), and main hosts (birds, horses), allowing for the prompt implementation of mitigation measures for reducing the risk of viral transmission through blood transfusions and blood donations by implementing systematic individual blood donation nucleic acid amplification tests (NAAT) until the end of transmission season [26,27,28,36,37].

According to a recently published review [19], a total of 1145 WNV infection cases were diagnosed between 2012 and 2020, for an incidence rate ranging between 0.090 cases per 100,000 persons (95% confidence intervals [95%CI] % 0.068 to 0.118) to 1.009 per 100,000 persons (95%CI 0.930 to 1.092), with 42.5% of them developing WNND. The very high share of WNND cases over the total of notified of WNV infections, as well as the particularly high detection rates among blood donors (annual incidence rates ranging between 1.353 cases per 100,000 specimens, 95% confidence intervals [95%CI] 0.279–3.953, to 19.069; 95%CI 13.494 to 26.174) led the authors to suspect a substantial underreporting of milder cases. After a relatively reduced notification of new incident cases during calendar year 2020 and 2021 [38,39], summer season 2022 was characterized by the unprecedented peak of new diagnoses of WNV, WNF, and complicated WNND [40,41,42]. For instance, by end of August 2022, a total of 440 WNV infections had been officially reported (49.0% of them characterized as WNND, 33.9% WNF), with 24 deaths [43].

Despite the increasing relevance of WNV as a human pathogen [8], and promising results from veterinary vaccines [44], neither human vaccines nor specific antiviral treatments have been licensed to date [45,46], and treatment options remain currently limited to the symptomatic care, benefiting from early and proper diagnosis from involved medical professionals [17,23]. As the physicians’ understanding of a certain disorder is also critical in modeling the acceptance of clinical options, including preventive practices where available [32,47,48], we specifically inquired a sample of Italian medical professionals on their understanding of WNV infections and tentative vaccines. Our aim is to characterize whether the extensive knowledge gaps that were previously identified [48] in the general population from endemic areas [32], as well as in a sample of occupational physicians [48], were also shared by medical professionals that could potentially care for incident WNF infection cases.

## 2. Materials and Methods

### 2.1. Study Design

We designed the present study as a cross-sectional online survey according to the STROBE statement (Strengthening the reporting of observational studies in epidemiology; see STROBE checklist as Supplementary Materials Table S1). The questionnaire was delivered through three closed medical discussion groups between 1 August 2022 and 10 September 2022. The aforementioned groups belonged to the Facebook Community “Memedical” that was founded during the SARS-CoV-2 as a medical mutual help community for medical professionals from the whole of Italy [49]. In total, after removing duplicated profiles, the targeted groups had 8800 unique members by 12 September 2022, encompassing medical professionals from various specialties and subspecialties, as well as medical settings (primary care, hospitals, etc.), with being a registered Italian medical professional the only requirement for eventual admission. 

The link to the questionnaire (Google Forms; Google LLC; Menlo Park, CA, USA) was posted into the discussion groups by the chief researcher (MR), having preventively contacted the group administrators and obtained their authorization. Participation in the study was voluntary, and the first page of the questionnaire contained full informed consent information: participants were guaranteed about the anonymous design of the survey, and that no personal data (e.g., name, IP address, email address, or any personal information unnecessary to the survey) would be requested, saved, or tracked. No financial compensation was offered to the respondents, but they were preventively informed that at the end of the questionnaire a full explanation of all items would be provided, representing an educative opportunity on WNV/WNF/WNND.

### 2.2. Inclusion Criteria

Participation was offered to all of the participants from the discussion group, but only individuals formally agreeing with the participation were able to complete the survey, being eventually included in the initial sample. To be included in the sample, the respondents were supposed to be: (1) qualified medical professionals (irrespective of their subspeciality); (2) living and working in Italy by summer 2022; (3) having any previous knowledge of WNV/WNF/WNND (i.e., “Have you any knowledge of the terms West Nile Virus/West Nile Fever?”). Inclusion criteria were assessed by a dichotomous question (yes vs. no) that was self-reported and not externally validated. Only individuals fulfilling inclusion criteria were retained for the final analyses. 

### 2.3. Sample Size

According to a previous study on knowledge, attitudes, and practices (KAP) of Italian medical professionals on WNV [48], an expected prevalence of 8.6% for having had any previous interaction with WNV during daily practice, a Type I error of 5% (0.05), and a power of 95% were cautiously assumed. The minimum sample size was therefore calculated as follows:

N = 1.96^2^ × 0.086 × (1 − 0.086)/0.05^2^ = 3.8416 × 0.086 × 0.914/0.0025 = 121

### 2.4. Questionnaire

The questionnaire is available as Appendix A Table A1, and included the following sections:

1. Main demographic data: age, gender, seniority as medical professional; the Italian region where the professional mainly worked and lived dichotomized as high-risk regions of the Po River Valley (i.e., Piedmont, Lombardy, Veneto, Emilia-Romagna, Friuli-Venezia Giulia) vs. all others. All questionnaires that lacked one or more demographic information were excluded from the analyses.
2. Knowledge test. Participants received specifically designed knowledge tests whose items were derived from an accurate analysis of available literature on WNV/WNF/WNND, including a total of: (a) 14 true/false statements (e.g., “WNF is caused by a virus that was only recently identified”; FALSE); (b) 4 multiple choice questions (e.g., “Case Fatality Ratio for WNF in European Union is usually estimated in…”; available options: 0.1%, TRUE; 1%; 10%; don’t know). A summary Knowledge score (GKS) was then calculated as a sum score, by adding +1 for every correct answer. On the contrary, wrong indications or a missing/“don’t know” answer added 0 to the summary GKS (potential range 0 to 22).
3. Risk perception. Risk perception can be defined as a function of the perceived probability of an event and its expected consequences. Therefore, participants were requested to rate, through a 5-point Likert scale, the perceived frequency (F^WNV^; “extremely infrequent”, score = 1; “infrequent”, score = 2; “neutral”, score = 3; “frequent”, score = 4; “very frequent”, score = 5) and the perceived severity (C^WNV^; “not at all severe”, score = 1; “low severity”, score = 2; “neutral”, score = 3; “severe”, score = 4; “very severe”, score = 5) of WNV infections in Italian population. Summary risk perception score (RPS) was therefore calculated as the product of F^WNV^ and C^WNV^ (potential range 1 to 25).
4. Attitudes. Respondents were requested to rate, through a full Likert scale of 1 (totally disagree) to 5 (totally agree), whether (a) they perceived WNV infections as a likely occurrence during daily activities in second half of 2022 and 2023 or not; (b) incident cases of WNV would affect their daily activities in second half of 2022 and 2023. Eventually, (c) they were similarly inquired about their confidence on being able or not to recognize a case of WNV infection. Participants were then asked to score (range: 1—not difficult to 10—very difficult) the perceived potential burden of WNV/WNF/WNND on the Italian National Health Service, alongside a series of selected disorders, namely seasonal influenza virus infection (SIV), SARS-CoV-2 infection, hepatitis B virus infection (HBV), tuberculosis (TB), and human immunodeficiency virus infection (HIV).
5. Practices. Participants were requested to report whether they had previously managed any case of WNV infection in the previous years (yes vs. no) or had previously received any university-level formation on WNV (yes vs. no), SIV vaccine during previous influenza season (i.e., 2021), or SARS-CoV-2 vaccine (at least 2 doses).
6. Vaccine propensity. To date, vaccines specifically targeting WNV are not commercially available. However, we assessed participants about their acceptance of a hypothetical WNV vaccine, and more precisely (a) whether they were favorable to receive a WNV vaccine when available, (b) how important they perceived the capability of the WNV vaccine to avoid natural infection, and (c) complications such as WNF and WNND. All attitudes were reported in a full scale of 1 (totally disagree) to 5 (totally agree). Participants were eventually inquired about their willingness to pay for a tentative WNV vaccine. More precisely, participants were requested to report how much they would accept to pay for a WNV vaccine, and the optimal price for a WNV vaccine) in the general population.

### 2.5. Ethical Considerations

The study was conducted according to the guidelines of the Declaration of Helsinki. A preventive ethical review and approval were waived for this study because of its anonymous, observational design and due to the lack of clinical data about patients that could configure the present research as a clinical trial. Participants were also guaranteed that retrieved data would be stored only for the time required by data analysis. The study, therefore, did not configure itself as a clinical trial, and a preliminary evaluation by an Ethical Committee was not required, according to Italian law (Gazzetta Ufficiale no. 76, dated 31 March 2008).

### 2.6. Data Analysis

Internal consistency of the questionnaire (i.e., the extent to which items within an instrument consistently measure the same characteristic or construct) was initially measured through calculation of the Cronbach’s alpha on the knowledge test as a scale of its reliability. A score ≥ 0.7 was considered for an “acceptable” internal consistency.

As a preliminary stage of data analysis, both GKS and RPS were normalized to percent values, then dichotomized by median value in high vs. low estimates. Likert scales were similarly dichotomized (i.e., score 4 and 5 vs. 1 to 3).

Descriptive analysis was performed as follows. Continuous variables were reported as average ± SD being initially tested for normal distribution by means of D’Agostino and Pearson omnibus normality test (cut-off value *p* < 0.100 for rejecting normal distribution). Normally distributed continuous variables were compared by means of Student’s *t*-test or ANOVA, where appropriate, while not normally distributed continuous variables were compared through Mann–Whitney or Kruskal–Wallis tests for multiple independent samples. Similarly, association between continuous variables was assessed through Pearson’s correlation coefficient (normally distributed variables) or Spearman’s rank correlation coefficient (not normally distributed variables).

Categorical values were reported as proportions and percent values, and their association with the reported risk perception (high vs. low) was assessed through chi-squared test.

All categorical variables that in univariate analysis were associated with higher risk perception having a *p* value < 0.100 were included in a multivariable model of binary logistic regression analysis in order to calculate their adjusted odds ratios (aOR) and respective 95% confidence intervals (95%CI).

All statistical analyses were performed by means of IBM SPSS Statistics 26.0 for Macintosh (IBM Corp. Armonk, NY, USA).

## 3. Results

### 3.1. Descriptive Analysis

**Figure 1:**
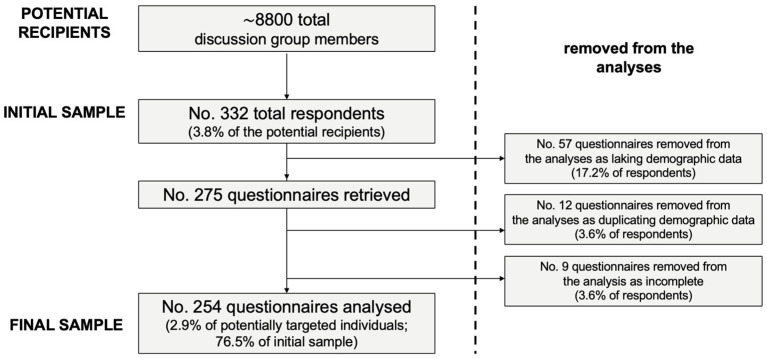
Flow chart of the selection of study participants.

A convenience sample of 332 medical professionals (3.8% of the potentially eligible population) eventually participated into this study. Of them, 254 fulfilled inclusion criteria and delivered a fully completed questionnaire, being included in the eventual analyses (Figure 1).

Briefly (Table 1), the majority of respondents were of female gender (53.5%), with a mean age of 38.2 years ± 9.5 (31.5% ≥ 40-year-old), while average seniority was 12.0 years ± 9.9 (52.0%% ≥ 10 years). Of them, 52.4% were from the endemic area of the Po River Valley.

### 3.2. Knowledge Test

Around 90.9% of participants reported any knowledge of WNV/WNF/WNND anticipating summer season 2022: in fact, 38.2% had reportedly received any university-level formation about this specific topic, while only 7.1% had any previous professional interaction with cases of WNV/WNF/WNND. 

**Figure 2:**
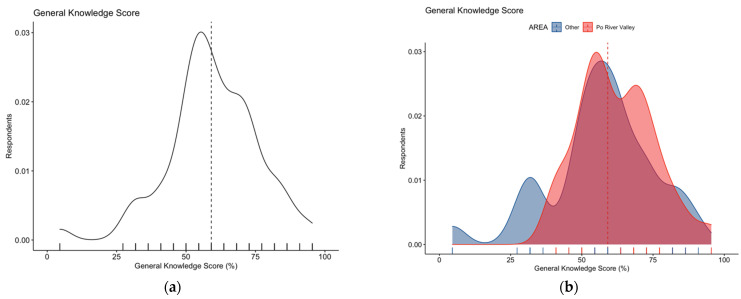
Density plots for: (**a**) general knowledge score in 254 Italian physicians participating into the survey; (**b**) general knowledge score broken down by residence of the respondents. The estimate was substantially skewed (D’Agostino–Pearson’s normality test skewness = −0.469, z = −2.993, *p*-value = 0.003), with higher score for participants residing vs. those not residing in endemic areas (62.2% ± 13.2 vs. 56.7% ± 18.2, Mann–Whitney *p* value = 0.038). Dotted line represents median values.

Focusing on the knowledge test, its internal consistency coefficient amounted to Cronbach’s alpha = 0.715, suggesting an acceptable reliability. After percent normalization, an unsatisfying GKS estimate of 59.6% ± 16.0 (actual range 4.6% to 95.5%, median 59.1%) was calculated. The distribution of the cumulative score did not pass the normality check (D’Agostino–Pearson normality test, *p* < 0.001) (Figure 2a), with substantial differences between individuals residing in the endemic vs. non-endemic area (62.2% ± 13.2 vs. 56.7% ± 18.2, Mann–Whitney *p* value = 0.038; Figure 2b).

The detailed results of the knowledge test are reported in Table A2. When focusing on the main knowledge gaps, substantial uncertainties were identified on the actual epidemiology of WNV infections, as only 14.6% of participants were aware that since 2010 WNV has been identified—in humans and/or animals—in the majority of Italian regions (Q15). Similarly, the large majority of participants improperly associated WNV infections with travels overseas (Q6; correct answers, 33.9%), while around half of respondents were aware that around 1000 cases of WNND had been identified in Italy during the last decade (Q10; 48.8%); that large mammals do not represent the main hosts for WNV (Q5; 49.2%), being rather dead-end hosts, like humans; and that usual case fatality ratio for WNV infections do not exceed 0.1% (Q14; 45.3%). Substantial misconceptions also affected the requirements for a proper diagnosis of WNV infections. On the one hand, respondents were somehow overconfident on serological tests, with 35.8% of respondents only being able to recognize the potential failure of serological tests in discriminating between various flavivirus infections (Q16). On the other hand, only half of the respondents were aware of official clinical recommendations following the recent European Case definition, which included fever as a not mandatory requirement for WNF diagnosis (Q19; 50.8%), and that flaccid paralysis represents a main diagnostic criterion for WNF and WNND (Q20; 48.8% of participants). Moreover, only 40.6% of respondents had any knowledge that the incubation of WNF may exceed 48 h from the initial infection (Q9). Interestingly, the majority of respondents were able to properly characterize a “confirmed” case (Q21; 68.5%), with greater uncertainties in the definition of a “probable” case (Q22; 48.8%).

### 3.3. Risk Perception

**Figure 3:**
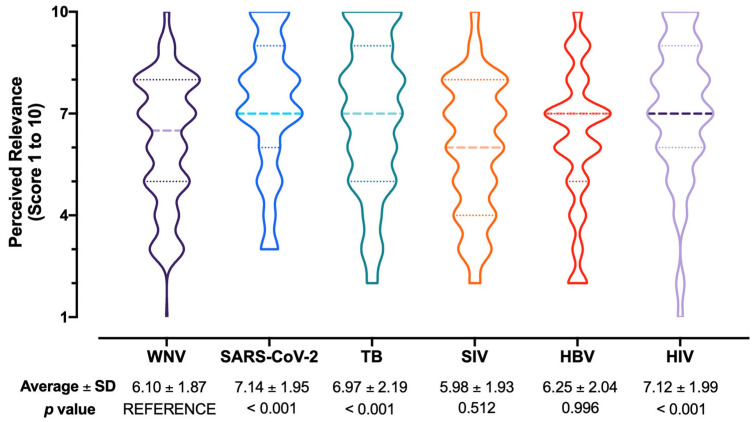
Box and violin plot for the perceived burden on National Health Service of West Nile virus infections (WNV) compared to SARS-CoV-2, tuberculosis (TB), seasonal influenza virus (SIV), hepatitis B virus (HBV), and human immunodeficiency virus (HIV) infections. Comparisons were performed by means of the Kruskal–Wallis test for multiple comparisons by assuming WNV/WNF/WNND as the reference group.

Nearly half of respondents (50.2%) perceived WNV/WNF/WNND as a likely occurrence during daily activities for the second half of 2022 and calendar year 2023, but a very low share (i.e., 16.1%) of participants were confident to be able to properly recognize a case of WNV/WNF/WNND. Still, the majority of respondents seemingly acknowledged WNV infections as not significant issue, as only one-fifth (20.2%) perceived disorders associated with WNV as potentially affecting their daily duties. Not coincidentally, the health threat represented by WNV/WNF/WNND on the National Health Service was rated through an overall score of 6.10 ± 1.87 (potential range 1 to 10, actual range 1 to 9 median 6.5; D’Agostino–Pearson K2 = 22.08, *p* < 0.001; Figure 3), for an estimated burden that was substantially lower than those associated with SARS-CoV-2 (7.14 ± 1.95, *p* < 0.001), TB (6.97 ± 2.19, *p* < 0.001), and HIV (7.12 ± 1.99, *p* < 0.001), while it was comparable to the scores for SIV (5.98 ± 1.93, *p* = 0.512) and HBV (6.25 ± 2.04, *p* = 0.996).

**Figure 4:**
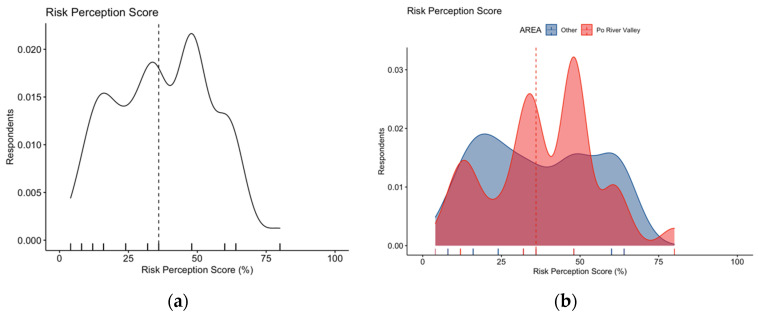
Density plots for: (**a**) risk perception score in 254 Italian physicians participating in the survey; (**b**) risk perception score broken down by residence of the respondents. The estimate was substantially skewed (D’Agostino–Pearson’s normality test *p*-value < 0.001), with no differences in score estimates for participants residing vs. those not residing in endemic areas (38.4% ± 17.0 vs. 36.9% ± 18.1 for individuals from Po River Valley vs. other Italian regions, Mann–Whitney *p* value = 0.644). Dotted line represents median values.

Focusing on RPS estimates, WNV infections were reported as either frequent or very frequent by 17.7% of respondents, while nearly half of participants (48.4%) reportedly acknowledged WNV infections as potentially severe or very severe. Eventually, an average score for RPS equals to 37.7% ± 17.5 was calculated (actual range: 4.0% to 76.0%, median 36.0%). A skewed distribution for RPS was both visually and statistically identified (Figure 4a, D’Agostino–Pearson test K2 = 23.85, *p* value < 0.001), with no substantial differences associated with the residence area (Figure 4b; 38.4% ± 17.0 vs. 36.9% ± 18.1 for individuals from the Po River Valley vs. other Italian regions; Mann–Whitney *p* value = 0.644).

### 3.4. Attitudes and Practices towards a Hypothetical WNV Vaccination

The majority of respondents exhibited a high or very high acceptance of recommended vaccinations for healthcare workers, as 96.1% had been allegedly vaccinated against SARS-CoV-2 during 2021, and 88.6% against SIV in the previous influenza seasons. 

In total, the majority of respondents (65.4%) were either favorable or highly favorable towards receiving a hypothetical WNV vaccine when made available. Focusing on the design of this vaccine, 88.6% identified its capability to avoid complications as significant/very significant, while 71.3% stressed the importance of avoiding natural infections. When dealing with the reported willingness to pay for a WNV vaccine, around 1/5 of participants (20.1%) were reportedly “not interested”, while 16.5% rated a total cost of EUR <10 per shot as somehow “acceptable”, 37.9% would consider a total cost ranging between EUR 10 and 49 per shot as adequate, 11.8% would consider a cost ranging from EUR 50 to 100 per shot, and 13.8% would agree to pay EUR 100 or more per shot. Regarding the optimal price for the general population, the majority of respondents reported that it should be either offered at no cost or with a cost of EUR <10 per shot for the recipients (57.1%). Interestingly, 39.4% recommended a cost ranging from EUR 10 to 49 per shot, while only 3.2% of participants identified as acceptable a payment ranging from EUR 50 to 100 per shot, and no one agreed for a total payment exceeding EUR 100 per shot.

### 3.5. Univariate Analysis

Interestingly, GKS and RPS were each other positively correlated (Spearman’s rho = 0.156; *p* value = 0.013), and in univariate analysis for dichotomous variables (Table 2), higher risk perception on WNV infections was positively associated with reporting a better GKS (57.9% vs. 31.4% among participants with a lower RPS, *p* < 0.001). Similarly, higher RPS was associated with seniority ≥ 10 years at the time of the survey (58.8% vs. 46.4%, *p* = 0.050), having had any previous interaction with WNV infections (12.3% vs. 2.9%, *p* = 0.004), and reporting a better attitude towards a hypothetical WNV vaccine (76.3% vs. 56.4%, *p* = 0.001). Similarly, perceiving WNV infections as a likely daily occurrence (58.8% vs. 46.4%, *p* = 0.050), potentially having a substantial impact of WNV cases on daily activities (29.8% vs. 12.1%, *p* < 0.001), and being confident to be able to recognize a WNV case (23.7% vs. 10.0%, *p* = 0.003), were more frequently reported among respondents scoring a higher RPS than among those with a low RPS.

### 3.6. Multivariable Analysis

Multivariable analysis was assessed through a binary logistic regression model that included higher RPS on WNV infection as the outcome variable, and the following explanatory variables: seniority ≥ 10 years; higher GKS; having any previous experience on WNV infection cases, reporting a better attitude towards a hypothetic WNV vaccine; perceiving WNV infections as potentially affecting their working activities; being confident to be able to recognize a WNV infection case; perceiving WNV infections as a likely occurrence during daily activities. 

As shown in Table 3, higher risk perception was positively associated with higher seniority (aOR 2.39, 95%CI 1.34 to 4.28), reporting a better knowledge status (aOR 2.90, 95%CI 1.60 to 5.30), having previously managed cases of WNV infections (aOR 3.65 95%CI 1.14 to 14.20), being favorable towards a hypothetic vaccine (aOR 2.12, 95%CI 1.15 to 4.04), and perceiving WNV infections as potentially affecting daily activities (aOR 2.57, 95%CI 1.22 to 5.42).

## 4. Discussion

**Figure A1:**
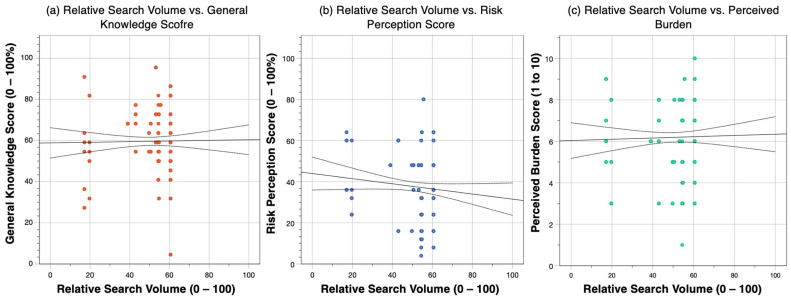
Correlation between relative search volumes (RSV) from Google Trends between 1 August and 10 September 2022 and: (**a**) general knowledge score (GKS, rho = −0.078, *p* = 0.217); (**b**) risk perception score (RPS, Spearman’s rho = −0.048; *p* = 0.451); (**c**) perceived burden of disease associated with West Nile virus infections (rho = 0.051; *p* = 0.419).

In the present cross-sectional study performed on 254 Italian medical professionals during summer season 2022, WNV infections were associated with a relatively unsatisfying knowledge status (with a potential range 0 to 100%, actual GKS was estimated to an average of 59.6% ± 16.0). Risk perception and knowledge status were not only correlated (Spearman’s rho = 0.156; *p* value = 0.013), but scoring a better knowledge status was identified as a positive effector for reporting higher risk perception (aOR 2.92, 95%CI 1.60 to 5.30). Still, even though the present study was performed during an unprecedent reporting season for WNV [41,42], participants were characterized by a relatively low risk perception (RPS 37.7% ± 17.5). In fact, WNV infections were generally considered as a lesser threat for public health than more “conventional” pathogens such as *Mycobacterium tuberculosis* and HIV, as well as SARS-CoV-2. Even though the potential burden of WNV infections was perceived akin to that associated with an important pathogen such as seasonal influenza, the latter is often underscored as a relatively indolent disorder, not only in the general population but also among Italian medical professionals [50,51,52], and the correspondent vaccine has been in turn often discredited by media [53,54,55,56,57,58,59]. A possible explanation may be found in the coverage of conventional and new media on the ongoing WNV outbreak in Northern Italy [40,41,42]. While an accurate measurement of the qualitative and quantitative coverage of a specific topic by conventional media remains particularly difficult to achieve, with obvious consequences on the eventual modeling of knowledge, beliefs, and perceptions of targeted study group, a proxy for new media has been developed and provided by Google^TM^ through Google Trends™ and the calculation of relative search volumes (RSV). Google Trends^TM^ is an open online tool developed by Google^TM^ for reporting web interest in a specific keyword or search topic [60,61,62]. Research interest is reported as RSV, which is a normalized value ranging from 0 to 100 and proportional to the ratio between the keyword-related queries and the total of web queries. Therefore, in order to rule out the potential bias represented by the overreporting of WNV in conventional media, we assessed whether any correlation did exist between knowledge status and risk perception on the one hand, and RSV on WNV from 1 August to 10 September 2022 (i.e., the exact timeframe where the web survey was made available) on the other hand. Eventually, during the study period, no substantial correlation with RSV on WNV was found for RPS (Spearman’s rho = 0.048; *p* = 0.451), GKS (rho −0.078, *p* = 0.217), and even for the perceived disease burden (rho = 0.041, *p* = 0.419 Figure A1) [61,62]. In other words, knowledge status, risk perception, and perceived disease burden of study participants were not seemly influenced by media coverage.

In fact, higher risk perception was also associated with individual factors such as reporting a seniority ≥ 10 years (aOR 2.39, 95%CI 1.34 to 4.28), having previously managed cases of WNV infections (aOR 3.65, 95%CI 1.14 to 14.20), being favorable towards a hypothetic vaccine (aOR 2.16, 95%CI 1.15 to 4.04), and perceiving WNV infections as potentially affecting daily activities (aOR 2.57, 95%CI 1.22 to 5.42). Interestingly enough, the majority of respondents was either favorable or even highly favorable to receiving a hypothetical WNV vaccine, but the share of uninterested respondents was substantial (around 1/5 of the whole sample). As an effective vaccine against WNV is still unavailable [46,63], having inquired the attitudes towards a tentative vaccine may appear somehow marginal to the main objectives of the present study (i.e., assessing KAP of Italian medical professionals on WNF infections), but there is an extensive base of evidence that a more positive attitude towards a certain vaccine is in turn associated with a better understanding of the prevented infectious diseases, and a more appropriate awareness of potential issues associated with communicable disorders [47,64,65]. Also in our study, exhibiting a positive attitude towards a tentative vaccine was characterized as a substantial affector of higher risk perception, being not only associated with higher knowledge status (See Table A3), but also with a proactive attitude towards immunizations for other infectious diseases such as SIV and SARS-CoV-2.

When dealing with our results, it should be stressed that the present study was deliberately designed in order to assess the actual understanding of epidemiological, clinical, and diagnostic features of WNV infections among Italian healthcare providers. Therefore, we did not inquire of study participants about common behavioral and environmental preventive options [32,48], whose knowledge and eventual acceptance has been previously inquired about in previous reports targeting both general population and selected occupational groups, including Italian occupational physicians [48,66,67,68,69]. This design was motivated by the lack of similar studies on medical professionals, and particularly from endemic areas of continental Europe. Even though WNV has progressively become an endemic pathogen to Northern Italy, and particularly to the Po River Valley [27,28,30,42,70,71,72,73], the actual understanding of WNV infections among Italian healthcare providers has been only scarcely inquired. Shortly before the inception of SARS-CoV-2 pandemic, a KAP study on 174 Italian occupational physicians [48] identified a relatively satisfying understanding of WNV infections and their preventive measures, still stressing the extensive underestimation of their actual occurrence in the general population and their severity as well. Despite a similar working definition for the risk perception, medical professionals participating into the present study reported a substantially higher risk perception on the potential severity of WNV infections, which was acknowledged as a severe one by 48.4% of participants compared to 8.6% of the previous survey. On the contrary, while in 2019 around 30.5% of respondents acknowledged WNV infections as frequent or highly frequent, the correspondent share dropped to 17.7% in the present study. Some explanations may be found in the characteristics of targeted professionals [74,75], as the individual expertise of occupational physicians—medical professionals primarily involved in health surveillance and preventive interventions in the workplaces—can be hardly compared to that of other medical professionals [47,48,75,76,77]. Another substantial factor could be identified in the decrease in notification rates for WNV infections between 2020 and 2021 [19,38,39], which was then followed by the unprecedented reporting season 2022, which was also characterized by a particularly high case fatality ratio [40,41,42]. In other words, after two reporting seasons with a low or even a very low circulation of the pathogen, participants may have been led to the perception of WNV as a relatively uncommon disorder, while the high lethality reported in 2022 could have inflated the perceived severity of WNV infections.

Not coincidentally, higher risk perception was associated with reporting greater seniority and with having any previous experience in the management of WNV infection cases. This latter remark is both consistent with some previous KAP studies on WNV infections [32,48,67,69] and with the underlying Health Belief Model (HBM) [78,79]. HBM was originally developed in the 1950s but remains one of the most widely used theories in health behavior research [80,81,82]. Its basic assumption is that the beliefs about the susceptibility to a certain health threat, correspondent perceptions on the potential severity of that threat, and perceived benefits (and conversely, barriers) associated with a particular intervention will determine whether or not an individual would adopt that action [83,84]. Interestingly enough, usual proxies for the better understanding of health threats and appropriate preventative options, such as the acceptance of certain vaccinations (e.g., SIV and SARS-CoV-2), were unrelated with higher risk perception for WNV infections [85,86,87,88]. Conversely (see Appendix A Table A3), both practices were associated with a favorable attitude towards a tentative WNV vaccine, alongside any previous interaction with WNV infections, which is consistent with HBM and currently acknowledged models for antecedents of vaccinations [65,80,81].

*Limits*. Despite its potential interest, the present article is affected by several limitations. First of all, the overall sample is quite small when compared not only to the whole of the Italian medical workforce (i.e., around 650,000 individuals in 2019) [89] but also to the potentially targeted medical professionals, as it included only 2.9% of individuals participating into the parent discussion group. Even though we were able to collect a total of 254 participants, potentially satisfying initial estimates for a minimum sample [48], the present study was hardy generalizable, for several reasons. For one, the sample size was calculated on the only KAP study on WNV infections previously performed on Italian medical professionals, but it only included occupational physicians, whose familiarity with WNV is hardly comparable with that of other caregiver workplaces [90,91], and whose representativity of the general medical workforce may be also reasonably questioned [47,91,92,93]. Moreover, Italy is characterized by distinctive regional patterns in background healthcare settings, and even in school-specific training during the residency programs [88]; as our study was characterized by a certain oversampling of participants from areas considered at higher risk for WNV infections, a potential overestimation of perceived burden of disease and risk perception cannot be ruled out. 

Still, as our study was performed during an unprecedented outbreak of WNV infections in the whole of the country [19,40,41,42], the present study may contribute to our understanding of baseline knowledge, attitudes and practices of medical professionals involved in the early identification and monitoring of this potentially serious infectious disease. As the large majority of WNV infections cases may occur without suggestive clinical signs and symptoms [17,23,94,95,96], assessing the actual understanding of WNV among healthcare providers may contribute to a better appreciation of the effective burden of disease in the general population [8,17,18,97].

Second, the present survey was designed as a web-based one, and this design is notably affected by several shortcomings, most notably including the potential “self-selection” of participants [98,99], and particularly those having greater familiarity with the Internet and social media, and more easily sharing personal information through the Internet and social media. In this regard, the mean age of our final sample was well under 40 years, with a reduced share of respondents aged 50 year or older, and these estimates are quite inconsistent with the demographics of the Italian medical workforce [89,100]. It is therefore reasonable that having targeted medical professionals participating in an internet discussion group may have led to the preventive selection of younger individuals that may fail to be representative of all Italian medical professionals, urging a very cautious interpretation of our results in more general terms. Likewise, having inquired a very specific topic (i.e., West Nile virus infection), the potential oversampling of subjects that were more familiar with the assessed topic than those not participating into the study is quite reasonable [98], as suggested by similarly designed cross-sectional studies [51,93,101]. 

Third, as the questionnaire was not externally validated, we cannot rule out that some of the respondents did not fully adhere to our inclusion, with a further impairment in the representativity of the sample. Nonetheless, discussion groups involved in the recruitment of the study participants did perform a preventive selection of their members by only including qualified medical professionals and eventually improving the reliability of our sample, at least when dealing with two of three inclusion criteria (i.e., being a licensed medical professional, and living and working in Italy).

Fourth, we cannot rule out that our results may have been influenced by some shortcomings in the implementation of current evidence in the Italian guidelines for WNV/WNF/WNND surveillance. More precisely, the Italian case definition of WNF has been recently revised and implemented in the Italian National Plan for the Prevention, Surveillance, and Response to Arboviruses 2020–2025 [102], consistently with the updated EU case definition [103]. Notably, the revised case definition acknowledged that fever does not represent an invariable and necessary feature of WNF [104], but only half of participants had any awareness that WNF may occur without noticeable fever. As the corresponding item in the knowledge test is clearly counter-intuitive, it possibly reflects the actual understanding of this topic by participants, that in turn may represent a lack of knowledge on the current medical evidence, or the misunderstanding of official guidelines. Interestingly, while the current EU case definition includes as clinical criteria for WNV infection at least one of the following three [103]: fever, encephalitis, and meningitis, correspondent Italian guidelines seemingly prioritize fever over encephalitis, meningitis, neuritis, and acute flaccid paralysis, the latter being considered alternative to the primary feature of fever [102].

Fifth, it is important to stress that the epidemiology of WNV in Italy should be ascertained in the more complex framework of flavivirus infections, whose understanding may be scarcely appreciable for professionals not involved in the prevention of infectious diseases, and particularly when dealing with laboratory diagnosis [2,19,39,105,106]. Flavivirus are structurally quite similar, which leads to some crossreactivity upon infection [107]. In Italy, viral pathogens such as Zika, dengue and yellow fever viruses are mostly associated with travel-related infections [108,109,110,111], but autochthonous transmission of dengue has recently occurred in the Veneto Region [112], and cocirculation of WNV and TBEV in the same Italian regions has been documented [19,106,108]. Even though a substantial share of diagnoses are based on serology, medical professionals should be aware that the very same National Guidelines warn about the potential impact of previous interactions with other flaviviruses, either as previous infections or vaccinations, recommending the preferential referral to molecular diagnostics (i.e., nucleic acid amplification test or NAAT) in certain settings, e.g., blood donors from high-risk areas [102]. As a consequence, participants with a limited understanding of these features of flavivirus infections may have improperly underestimated the potential lack of specificity of serology in Italian settings (and particularly in the Veneto Region, where a potential cocirculation of WNV, dengue virus, and TBEV cannot be ruled out a priori) [29,108,113]. On the other hand, international readers should be particularly careful in the generalization of this item of the knowledge test and of its meaning, particularly in settings where WNV is the main or even the sole circulating flavivirus.

Eventually, despite the knowledge score and risk perception score being seemingly unaffected by background media coverage on the ongoing WNV outbreak, our estimates should be acknowledged as strictly dependent on the exact timeframe of this study, while we cannot rule out that a follow-up study would lead to a very different outcome.

## 5. Conclusions

The ongoing endemization of WNV in Western Europe urges an increased knowledge status and suspicion index of medical professionals on this pathogen. Our study identified an unsatisfying knowledge status and a relatively low risk perception of the Italian medical workforce on WNV infection. The main determinant of higher risk perception was identified in a better understanding of WNV infections, and having had any previous interaction with this pathogen, which is consistent with the HBM. Despite the implicit limits of this study, our methodology could be implemented for future studies monitoring the knowledge status of medical professionals towards WNV infections, allowing for a better targeting of knowledge gaps and false beliefs, eventually improving the capability of medical workforce to properly and actively identify new incident cases of WNV infections.

## Figures and Tables

**Table 1:** Characteristics of the 254 Italian medical professionals participating into the survey on knowledge, attitudes, and practices on West Nile virus (WNV) infections (Italy, Summer 2022).

Variable	No./254	Average ± SD
Gender		
Male	118, 46.5%	
Female	136, 53.5%	
Endemic area for WNV	133, 52.4%	
Age (years)		38.2 ± 9.5
Seniority (years)		12.0 ± 9.9
Any knowledge of WNV/WNF/WNND before 2022	231, 90.9%	
Previously managed cases of WNV infections	18, 7.1%	
Any university-level formation on WNV infections	97, 38.2%	
Acknowledging WNV infections as frequent/very frequent	45, 17.7%	
Acknowledging WNV infections as severe/very severe	123, 48.4%	
General knowledge score (%)		59.6 ± 16.0
General knowledge score > median (59.1%)	110, 43.3%	
Risk perception Score (%)		37.7 ± 17.5
Risk perception Score > median (36.0%)	114, 44.9%	
Favorable/highly favorable to WNV vaccination when made available	166, 65.4%	
Acknowledging as significant/very significant aspects for WNV vaccines		
Avoiding natural infection	181, 71.3%	
Avoiding complications	225, 88.6%	
Willingness to pay for a hypothetic WNV vaccine (himself)		
Not interested	51, 20.1%	
Free or <10 EUR/shot	42, 16.5%	
10–19 EUR/shot	29, 11.4%	
20–29 EUR/shot	30, 11.8%	
30–39 EUR/shot	7, 2.9%	
40–49 EUR/shot	30, 11.8%	
50–100 EUR/shot	30, 11.8%	
>100 EUR/shot	35, 13.8%	
Acceptance to pay for a hypothetic WNV vaccine (population)		
Free or <10 EUR/shot	145, 57.1%	
10–19 EUR/shot	22, 8.7%	
20–29 EUR/shot	50, 19.7%	
30–39 EUR/shot	17, 6.7%	
40–49 EUR/shot	12, 4.7%	
50–100 EUR/shot	8, 3.2%	
>100 EUR/shot	0, -	
Vaccinated against SARS-CoV-2 in 2021	244, 96.1%	
Vaccinated against seasonal influenza in previous years	220, 86.6%	
Perceiving WNV infections as potentially affecting working activities (agree/totally agree)	51, 20.1%	
Confident to be able to recognize a WNV infection case (agree/totally agree)	41, 16.1%	
Perceiving WNV infections as a likely occurrence during daily activity (agree/totally agree)	132, 52.0%	

**Table 2:** Characteristics of the 254 Italian medical professionals participating into the survey on knowledge, attitudes, and practices on West Nile virus (WNV) by having or not having higher risk perception on WNV infections.

Variable	Risk Perception on WNV		*p* Value
	High/Very High (No./114, %)	Low (No./140, %)	
Age ≥ 40 years	40, 35.1%	40, 28.6%	0.266
Seniority ≥ 10 years	67, 58.8%	65, 46.4%	0.050
Male gender	51, 44.7%	67, 47.9%	0.620
Living in endemic area	62, 54.5%	71, 50.7%	0.560
Higher knowledge status	66, 57.9%	44, 31.4%	<0.001
Any previous knowledge of WNV/WNF/WNND	106, 93.0%	125, 89.3%	0.307
University-level formation on WNV	38, 33.3%	59, 42.1%	0.151
Previously managed WNF cases	14, 12.3%	4, 2.9%	0.004
Favorable attitude towards a hypothetic vaccine	87, 76.3%	79, 56.4%	0.001
Vaccinated against SARS-CoV-2 in 2021	111, 97.4%	133, 95.0%	0.334
Vaccinated against seasonal influenza in previous years	99, 86.8%	121, 86.4%	0.923
Perceiving WNV infections as potentially affecting working activities (agree/totally agree)	34, 29.8%	17, 12.1%	<0.001
Confident to be able to recognize a WNV infection case (agree/totally agree)	27, 23.7%	14, 10.0%	0.003
Perceiving WNV infections as a likely occurrence during daily activity (agree/totally agree)	67, 58.8%	65, 46.4%	0.050

**Table 3:** Multivariable analysis of factors associated with having high risk perception WNV infections. Adjusted odds ratios (aOR) and their respective 95% confidence intervals were calculated through binary logistic regression analysis, including as explanatory variables all factors that in univariate analysis were associated with the outcome variables with *p* < 0.100.

Variable	High Risk Perception on WNV Infections	
	aOR	95% CI
Seniority ≥ 10 years	2.39	1.34; 4.28
Higher knowledge status	2.92	1.60; 5.30
Previously managed WNV cases	3.65	1.14; 14.20
Favorable attitude towards a hypothetic vaccine	2.16	1.15; 4.04
Perceiving WNV infections as potentially affecting working activities (agree/totally agree)	2.57	1.22; 5.42
Confident to be able to recognize a WNV infection case (agree/totally agree)	2.06	0.91; 4.66
Perceiving WNV infections as a likely occurrence during daily activity (agree/totally agree)	1.32	0.74; 2.35

Notes: aOR = adjusted odds ratio (i.e., odds ratio calculated through binary logistic regression); 95%CI = 95% confidence interval.

## Data Availability

The original questionnaire can be freely shared and modified by the end users (See Appendix A Table A1).

## Supplementary Materials

The following supporting information can be downloaded at: https://www.mdpi.com/article/10.3390/tropicalmed7120404/s1, Table S1: STROBE checklist. File S2: Author’s translation of the informed consent.

## Appendix A

**Table A1:** Authors’ translation of the items included in the questionnaire.

Section 1. Your Personal Experience with WNV Infections: during Your Clinical Practice	
Had you any knowledge of the terms WNV/WNF/WNND before summer 2022?	[YES] [NO] [NO ANSWER]
Have you previously managed any WNV infection case?	[YES] [NO] [NO ANSWER]
Did you receive any University-level formation on WNV infections?	[YES] [NO] [NO ANSWER]
Have previously required any hospitalization for RSV?	[YES] [NO] [NO ANSWER]
**Section 2. At Your Knowledge (Please Mark the Correct Answer)**	
1. The agent of West Nile Fever is a newly identified virus, discovered some months ago	[TRUE] [FALSE] [DON’T KNOW]
2. West Nile Virus has common interhuman spreading	[TRUE] [FALSE] [DON’T KNOW]
3. In most cases, WNV evolves in an uncomplicated influenza-like illness	[TRUE] [FALSE] [DON’T KNOW]
4. West Nile Fever is characterized by pathognomonic skin lesions	[TRUE] [FALSE] [DON’T KNOW]
5. Large mammals (e.g., horses) represent the main hosts of the WNV	[TRUE] [FALSE] [DON’T KNOW]
6. In Europe, the majority of cases occur following travels to endemic areas oversea	[TRUE] [FALSE] [DON’T KNOW]
7. An effective vaccine against WNV is commercially available	[TRUE] [FALSE] [DON’T KNOW]
8. Drugs specifically targeting WNV are commercially available	[TRUE] [FALSE] [DON’T KNOW]
9. Incubation of WNF is usually short, less than 48 h from the infection	[TRUE] [FALSE] [DON’T KNOW]
10. During the last 10 years, around … cases of WNND have been officially notified	
100 cases	[ ]
1000 cases	[ ]
10,000 cases	[ ]
Don’t know	[ ]
11. WNV infections are associated with systemic complications (e.g., meningitis, hepatitis, pancreatitis, myocarditis) in the majority of cases.	[TRUE] [FALSE] [DON’T KNOW]
12. WNV infections are less severe in children than in adults/elderly	[TRUE] [FALSE] [DON’T KNOW]
13. Point of care tests are effective options for the serological diagnosis of WNV infections	[TRUE] [FALSE] [DON’T KNOW]
14. In Western Europe, case fatality ratio for WNV infections is usually estimated to … (around)	
… 0.1%	[ ]
… 1.0%	[ ]
… 10%	[ ]
Don’t know	[ ]
15. Since 2010, WNV has been identified (in humans and/or animals) …	
… only in some areas (i.e., some municipalities and provinces)	[ ]
… in the majority of Italian regions	[ ]
… in some Italian regions	[ ]
Don’t know	[ ]
16. Serological tests for WNV are highly effective and specific	[TRUE] [FALSE] [DON’T KNOW]
17. Blood donations are usually monitored for WNV infections	[TRUE] [FALSE] [DON’T KNOW]
18. According to Italian National Guidelines, a case is considered a “probable” WNF case:	
Detectable antibodies with or without clinical criteria	[ ]
Detectable IgG with clinical criteria	[ ]
Detectable IgM with clinical criteria	[ ]
Clinical criteria only	[ ]
Don’t know	[ ]
19. Fever (body temperature > 38 °C) is necessary to the diagnosis of WNF	[TRUE] [FALSE] [DON’T KNOW]
20. Flaccid paralysis is acknowledged among clinical criteria for WNF	[TRUE] [FALSE] [DON’T KNOW]
21. A case characterized by: (1) fever (T > 38 °C), (2) signs of encephalitis (i.e., headache, altered mental state, with or without seizures and neurological issues); (3) residence in high risk areas (e.g., Veneto or Emilia Romagna) may be classified as a …	
… confirmed case	[ ]
… doubtful case	[ ]
… probable case	[ ]
Don’t know	[ ]
22. A case characterized by: (1) fever (T > 38 °C), (2) flaccid paralysis; (3) residence in high risk areas (e.g., Veneto or Emilia Romagna); (4) IgM specifically targeting WNV (unavailable dosage) may be classified as a…	
… confirmed case	[ ]
… doubtful case	[ ]
… probable case	[ ]
Don’t know	[ ]
23. Please rate the following items from “not significant” (1) to “very significant” (5)	
How do you perceive the frequency of WNV infections?	[1] [2] [3] [4] [5]
How do you perceive the severity of WNV infections?	[1] [2] [3] [4] [5]
24. From your point of view, how would you rate the potential burden on National Health Service of the following infections? (1 minimum to 10 maximum)	
WNV	[1] [2] [3] [4] [5] [6] [7] [8] [9] [10]
SARS-CoV-2	[1] [2] [3] [4] [5] [6] [7] [8] [9] [10]
Seasonal Influenza	[1] [2] [3] [4] [5] [6] [7] [8] [9] [10]
Tuberculosis	[1] [2] [3] [4] [5] [6] [7] [8] [9] [10]
Hepatitis B Virus infection	[1] [2] [3] [4] [5] [6] [7] [8] [9] [10]
HIV	[1] [2] [3] [4] [5] [6] [7] [8] [9] [10]
25. Are you favorable towards the implementation of a WNV vaccine in the specific vaccine schedule, if commercially available? (1 = totally disagree; 5 = totally agree)	[1] [2] [3] [4] [5]
26. In the use of a hypothetical vaccine against WNV, which aspects are of specific importance, from your point of view? (1 = totally disagree; 5 = totally agree)	
… avoiding natural infections	[1] [2] [3] [4] [5]
… avoiding complications (i.e., LRTI)	[1] [2] [3] [4] [5]
27. In case an effective vaccine against WNV would be made available, how much would you accept to pay for a single shot?	
Not interested	[ ]
Free or < 10 €/shot	[ ]
10–19 €/shot	[ ]
20–29 €/shot	[ ]
30–39 €/shot	[ ]
40–49 €/shot	[ ]
50–100 €/shot	[ ]
>100 €/shot	[ ]
28. In case an effective vaccine against WNV would be made available, how much would you retain as acceptable expense for the general population for a single shot?	
Not interested	[ ]
Free or < 10 €/shot	[ ]
10–19 €/shot	[ ]
20–29 €/shot	[ ]
30–39 €/shot	[ ]
40–49 €/shot	[ ]
50–100 €/shot	[ ]
>100 €/shot	[ ]
29. From your point of view… (1 totally disagree to 5 totally agree)	
… in the following months, WNV infections will affect your daily working activities	[1] [2] [3] [4] [5]
… in the following months, WNV will be a likely occurrence during daily activities	[1] [2] [3] [4] [5]
… you will be able to properly recognize a WNV infection case during daily activities	[1] [2] [3] [4] [5]
30. Please provide some general information about you	
Year of birth:	______________
Year of medical qualification:	______________
You identify yourself as:	[Male] [Female] [No Answer]
Do you work in hospital settings?	[yes] [no] [no answer]
Are you resident in any of the following Italian Regions?	
Piedmont, Lombardy, Veneto, Friuli-Venezia-Giulia, Emilia Romagna	[ ]
Other	[ ]

Notes: The present questionnaire can be shared and modified by the end user. Please cite the present paper.

**Table A2:** Knowledge test: summary of the answers on presented items proposed to the 254 Italian Medical Professionals who participated into the present survey on West Nile virus infections (Cronbach’s alpha = 0.707).

Statement	Correct Answer	Total (No./389)
Q01. The agent of West Nile Fever is a newly identified virus, discovered some months ago	FALSE	246, 96.9%
Q02. West Nile Virus has common interhuman spreading	FALSE	229, 90.2%
Q03. In most cases, WNV evolves in an uncomplicated influenza-like illness	TRUE	225, 88.6%
Q04. West Nile Fever is characterized by pathognomonic skin lesions	FALSE	195, 76.8%
Q05. Large mammals (e.g., horses) represent the main hosts of the WNV	FALSE	125, 49.2%
Q06. In Europe, the majority of cases occur following travels to endemic areas oversea	FALSE	86, 33.9%
Q07. An effective vaccine against WNV is commercially available	FALSE	218, 85.8%
Q08. Drugs specifically targeting WNV are commercially available	FALSE	156, 61.4%
Q09. Incubation of WNF is usually short, less than 48 h from the infection	FALSE	103, 40.6%
Q10. During the last 10 years, around … cases of WNND have been officially notified		
100 cases	FALSE	35, 13.8%
1000 cases	TRUE	124, 48.8%
10,000 cases	FALSE	73, 28.7%
Don’t know	-	22, 8.7%
Q11. WNV infections are associated with a high risk of systemic complications	FALSE	162, 63.8%
Q12. WNV infections are less severe in children than in adults/elderly	TRUE	135, 53.1%
Q13. Point of care tests are effective options for the serological diagnosis of WNV infections	FALSE	154, 60.6%
Q14. In Western Europe, case fatality ratio for WNV infections is usually estimated to … (around)		
… 0.1%	TRUE	115, 45.3%
… 1.0%	FALSE	112, 44.1%
… 10%	FALSE	16, 6.3%
Don’t know	-	11, 4.3%
Q15. Since 2010, WNV has been identified (in humans and/or animals) …		
… only in some areas	FALSE	37, 14.6%
… in the majority of Italian regions	TRUE	37, 14.6%
… in some Italian regions	FALSE	165, 65.0%
Don’t know	-	15, 5.8%
Q16. Serological tests for WNV are highly effective and specific	FALSE	91, 35.8%
Q17. Blood donations are usually monitored for WNV infections	TRUE	206, 81.1%
Q18. According to Italian National Guidelines, a case is considered a “probable” WNF case:		
Detectable antibodies with or without clinical criteria	FALSE	23, 9.1%
Detectable IgG with clinical criteria	FALSE	15, 5.9%
Detectable IgM with clinical criteria	TRUE	178, 70.1%
Clinical criteria only	FALSE	19, 7.5%
Don’t know	-	19, 7.5%
Q19. Fever (body temperature > 38 °C) is necessary to the diagnosis of WNF	FALSE	125, 50.8%
Q20. Flaccid paralysis is acknowledged among clinical criteria for WNF	TRUE	124, 48.8%
Q21. A case characterized by: (1) fever (T > 38 °C), (2) signs of encephalitis (i.e., headache, altered mental state, with or without seizures and neurological issues); (3) residence in high risk areas (e.g., Veneto or Emilia Romagna) and high level of IgM immunoglobulins with or without IgG may be classified as a …		
… confirmed case	TRUE	174, 68.5%
… doubtful case	FALSE	4, 1.6%
… probable case	FALSE	63, 24.8%
Don’t know	-	13, 5.1%
Q22. A case characterized by: (1) fever (T > 38 °C), (2) flaccid paralysis; (3) residence in high risk areas (e.g., Veneto or Emilia Romagna); (4) IgM specifically targeting WNV (unavailable dosage) may be classified as a…		
… confirmed case	FALSE	93, 36.6%
… doubtful case	FALSE	20, 7.9%
… probable case	TRUE	124, 48.8%
Don’t know	-	17, 6.7%

**Table A3:** Characteristics of the 254 Italian medical professionals participating into the survey on knowledge, attitudes, and practices on West Nile Virus (WNV) by being or not being favorable on receiving a tentative WNV vaccine.

Variable	Attitude towards a Tentative WNV Vaccine		*p* Value
	Favorable (No./166, %)	Not Favorable (No./88, %)	
Age ≥ 40 years	48, 28.9%	32, 36.4%	0.224
Seniority ≥ 10 years	82, 49.4%	50, 56.8%	0.260
Male gender	68, 41.0%	50, 56.8%	0.016
Living in endemic area	94, 56.6%	39, 44.3%	0.062
Higher knowledge status	88, 53.0%	22, 25.0%	<0.001
Higher risk perception status	87, 52.4%	27, 30.7%	0.001
Any previous knowledge of WNV/WNF/WNND	150, 90.4%	81, 92.0%	0.656
University-level formation on WNV	58, 34.9%	39, 44.3%	0.143
Previously managed WNF cases	10, 6.0%	8, 9.1%	0.365
Vaccinated against SARS-CoV-2 in 2021	163, 98.2%	81, 92.0%	0.017
Vaccinated against seasonal influenza in previous years	154, 92.8%	66, 75.0%	<0.001
Perceiving WNV infections as potentially affecting working activities (agree/totally agree)	41, 24.7%	10, 11.4%	0.012
Confident to be able to recognize a WNV infection case (agree/totally agree)	24, 15.1%	16, 18.2%	0.520
Perceiving WNV infections as a likely occurrence during daily activity (agree/totally agree)	89, 53.6%	43, 48.9%	0.471
