# Supplementary material for: West Nile Virus Infection: A Cross-Sectional Study on Italian Medical Professionals during Summer Season 2022
Source: Trop Med Infect Dis. 2022 Nov 28;7(12):404. doi: 10.3390/tropicalmed7120404 (PMC9786547; doi:10.3390/tropicalmed7120404)
Supplement: Supplementary file 1 [file tropicalmed-07-00404-s001.zip › tropicalmed-2020867-supplementary.pdf]

Supplementary Table S1. Strobe statement Checklist

	Item No	Recommendation	Page No
		**Title and Abstract**	
	1	(*a*) Indicate the study's design with a commonly used term in the title or the abstract	1
		(*b*) Provide in the abstract an informative and balanced summary of what was done and what was found	1
		**Introduction**	
Background/rationale	2	Explain the scientific background and rationale for the investigation being reported	1-3
Objectives	3	State specific objectives, including any prespecified hypotheses	2-3
		**Methods**	
Study design	4	Present key elements of study design early in the paper	3
Setting	5	Describe the setting, locations, and relevant dates, including periods of recruitment, exposure, follow-up, and data collection	3-4
Participants	6	(*a*) Give the eligibility criteria, and the sources and methods of selection of participants	3
Variables	7	Clearly define all outcomes, exposures, predictors, potential confounders, and effect modifiers. Give diagnostic criteria, if applicable	3-5
Data sources/ measurement	8*	For each variable of interest, give sources of data and details of methods of assessment (measurement). Describe comparability of assessment methods if there is more than one group	4-5
Bias	9	Describe any efforts to address potential sources of bias	4-5
Study size	10	Explain how the study size was arrived at	3, 6
Quantitative variables	11	Explain how quantitative variables were handled in the analyses. If applicable, describe which groupings were chosen and why	5
Statistical methods	12	(*a*) Describe all statistical methods, including those used to control for confounding	5
		(*b*) Describe any methods used to examine subgroups and interactions	5
		(*c*) Explain how missing data were addressed	3-5
		(*d*) If applicable, describe analytical methods taking account of sampling strategy	5
		(*e*) Describe any sensitivity analyses	18
		**Results**	
Participants	13*	(a) Report numbers of individuals at each stage of study—eg numbers potentially eligible, examined for eligibility, confirmed eligible, included in the study, completing follow-up, and analysed	6
		(b) Give reasons for non-participation at each stage	6
		(c) Consider use of a flow diagram	6
Descriptive data	14*	(a) Give characteristics of study participants (eg demographic, clinical, social) and information on exposures and potential confounders	6-7
		(b) Indicate number of participants with missing data for each variable of interest	6
Outcome data	15*	Report numbers of outcome events or summary measures	6-10
Main results	16	(*a*) Give unadjusted estimates and, if applicable, confounder-adjusted estimates and their precision (eg, 95% confidence interval). Make clear which confounders were adjusted for and why they were included	10
		(*b*) Report category boundaries when continuous variables were categorized	10-11
		(*c*) If relevant, consider translating estimates of relative risk into absolute risk for a meaningful time period	-
Other analyses	17	Report other analyses done—eg analyses of subgroups and interactions, and sensitivity analyses	8-11
		**Discussion**	
Key results	18	Summarise key results with reference to study objectives	11
Limitations	19	Discuss limitations of the study, taking into account sources of potential bias or imprecision. Discuss both direction and magnitude of any potential bias	13-14
Interpretation	20	Give a cautious overall interpretation of results considering objectives, limitations, multiplicity of analyses, results from similar studies, and other relevant evidence	11-13
Generalisability	21	Discuss the generalisability (external validity) of the study results	13

**Other Information**			
Funding	22	Give the source of funding and the role of the funders for the present study and, if applicable, for the original study on which the present article is based	15

*Give information separately for exposed and unexposed group.

Supplementary File S2. Author's translation of the informed consent

Estimated colleague, the present survey has been developed and shared with the aim to assess the knowledge of medical professionals on the West Nile Virus and its main complications (i.e. West Nile Fever and West Nile Neuro-invasive Disease). The present survey specifically targets professionals having a medical background and only has scientific aims. In order to thank you for your cooperation, at the end of the questionnaire (whose items are based on the available scientific evidence) the final score will be shown alongside the detailed answers to the reported questions. Participating into the present questionnaire, you will have a chance to update your understanding of the WNV infections (by end of July, 2022).

While we thank you for your cooperation, we stress that web-based surveys must fulfill the requirements represented by the "Helsinki protocol" and EU Regulation 2016/679.

In order to fulfill the requirements of the Helsinki protocol, we're requesting to formally share your consent. Without your consent, the survey will not continue. Even after your consent, you can leave the present survey at any moment, until the sharing of the questionnaire (button "share module" at the end of the questionnaire. Moreover, we stress that the questionnaire will be registered in anonymous form, and in no way it could be associated with the compiler, as we will not retain any specific, individual information (e.g., email address, or IP address of your computer). All requested personal data are generic ones, and functional to the demographic analyses (gender, age, etc.).

According to the EU Regulation 2016/279 (GDPR), we also state that:

1) data controller, processor, as well as responsible of their retention during the analyses will be Dr. ********** whom you can ask about the process through his personal email (*********). Collected data are generic ones, with SOLE SCIENTIFIC AIMES that have been previously reported. Please be aware that all personal data must be shared with Criminal Law Authorities, without a previous personal consent, in the cases that are specifically reported by the current legal framework, without a specific request, retrieved will not be shared with third parts.
2) after the completion of the questionnaire, we cannot identify in any way the compiler; as the questionnaire is totally anonymous by design, we cannot perform any modification, correction of data collected, and their removal as well.
3) Data will be retained only for the time strictly required for the aforementioned analyses.

Do you agree to participate into the present survey? YES / NO
